# Perceptions and Acceptability of a Smartphone App Intervention (ChildSafe) in Malaysia: Qualitative Exploratory Study

**DOI:** 10.2196/24156

**Published:** 2021-06-01

**Authors:** Teresa Sui Mien Yong, Komathi Perialathan, Masitah Ahmad, Nurashma Juatan, Liana Abdul Majid, Mohammad Zabri Johari

**Affiliations:** 1 Institute for Health Behavioural Research National Institutes of Health Ministry of Health Malaysia Shah Alam Malaysia; 2 Institute of Public Health National Institutes of Health Ministry of Health Malaysia Shah Alam Malaysia

**Keywords:** child safety, unintentional injuries, consolidated framework for implementation research (CFIR), characteristics of individuals, Mobile App Rating Scale (MARS)

## Abstract

**Background:**

Home is a vulnerable place for accidental child injuries. Unintentional injuries are a leading cause of death, hospitalization, and disabilities. These injuries are considered preventable and if not tackled, they will continue to be a persisting problem. Smartphones have become increasingly important in our everyday life and is an important tool not only for communication but also for other purposes—they have apps that can be used for various purposes. Therefore, an app-based intervention (ChildSafe) was developed to assess and reduce child injury at home.

**Objective:**

This study aimed to evaluate the acceptance of the ChildSafe smartphone app intervention by parents/guardians.

**Methods:**

This study was conducted using a qualitative exploratory approach on selected participants of the ChildSafe intervention app study. A total of 27 semistructured in-depth interviews were carried out among parents or guardians who have at least one child between the age of 0 and 59 months in the area of Sungai Buloh, Selangor, between November 2017 and March 2018. Interview questions were developed from the consolidated framework for implementation research (CFIR). Interviews were recorded, transcribed verbatim, and data were thematically analyzed guided by CFIR.

**Results:**

The study revealed users’ perception on usability, feasibility, and acceptability toward the ChildSafe app. Three CFIR domains were identified: intervention characteristics, inner setting, and characteristics of individuals. A total of 5 constructs were revealed under intervention characteristics: evidence strength and quality, relative advantage, adaptability, trialability, and design quality and packaging; 2 under inner setting: implementation climate and readiness for implementation; and 4 under characteristics of individuals: knowledge and beliefs about the intervention, self-efficacy, individual stage of change, and other personal attributes. In general, participants felt the app is extremely useful and effective, easy to use, and purposeful in achieving home safety assessment via reminders. The app replaces the need for participants to search for information on home safety and dangers, as the app itself was designed as a tool to assess for this specific purpose. Even at the nascent stage and despite its limitations, the app has prompted users to consider and make changes around their own home. However, future versions of the app should be expanded to make it more attractive to users as it lacks interactive feedback and additional features.

**Conclusions:**

Parents/guardians are accepting the use of the ChildSafe app to prevent child injury at home. However, further expansion and improvements are needed to increase the acceptability of this app by parents/guardians.

## Introduction

Home is where individuals or every family member feels secured and protected. Despite providing shelter and comfort, children can still be exposed to hazards and risks, and are vulnerable to unintentional injuries. Child injuries at home are one of the most common global public health threats to their survival [1]. Unintentional injuries refer to traffic injuries, drowning, poisonings, burns, and falls and are believed to be the leading cause of death, hospitalization, and disability globally and can cause long-lasting grief to the families, society, and nation [2].

After 15 years the Millennium Development Goals (MDGs) 4 still has not achieved the two-third reduction in under 5 mortality rate (U5MR) globally [3], with 5.82 million deaths reported among children under the age of 5 worldwide; injury-specific mortality rate in the under 5 age group was 73 per 100,000 population and 3654 years of life were lost per 100,000 population in 2015 [4]. The Malaysia National Health and Morbidity Survey revealed that the prevalence of home injury among children aged under 4 years was 2.5% in 1996, whereas the rate among children aged under 7 years was 8.2% in 2011 [5]_._

Despite preventive measures, concerted efforts are still needed from various agencies to reduce further childhood injuries and the overall related mortality and morbidity issues [4]. The 2011 World Health Assembly urged member states to elevate child injury as a priority in the global public health policy [4]. The World Report on Child Injury Prevention highlighted the urgent need to address preventable cause of death and disability among vulnerable children worldwide; the World Health Organization, the United Nations Children’s Fund, and other organizations have initiated joint effort initiatives to safeguard children’s rights to healthy and safe environment free from injury and violence as emphasized by the United Nations Convention on the Rights of a Child (UNCRC) [2,6,7].

Henceforth, Sustainable Development Goals (SDGs) 2016-2030 have included targets for injury and violence prevention. The third goal is to reduce the deaths of children under 5 years of age from 9.8 million in 2000 to 5.4 million by 2017 [8].

Parenting interventions can reduce incidents of unintentional injuries in child in and around the home through the use of safety equipment and following existing safety recommendations by focusing on improving knowledge and perceptions on risks of injury, and importance of adopting safety practices [9]. Gaines and Schwebel [10] highlighted that parents and caregivers often have difficulties in identifying and recognizing hazards and perceived their own children to have less vulnerability to injuries. Importance of educating parents to find credible information and recommendations, and obtaining safety products best suited to their home were recommended.

Various intervention methods had been used with varying degrees of effectiveness, such as provision of educational materials, health care provider counselling, safety product distribution, and hands-on experiential learning provided at safety resource centers, to prevent injuries at home. However, it accentuated the need for wide-reaching, effective, and readily available methods of interventions to reach substantial parent and caregiver audiences [11].

Academics and clinicians are keenly interested in utilizing smartphones as an option for delivery of behavioral interventions [12]. Smartphone has become an integral part of our lives as it allows us to perform certain tasks and enables getting information, entertainment, and staying connected with others. The smartphone market is growing rapidly, with estimates suggesting that there are now 3.5 billion users worldwide (45.15%), up from 2.5 billion (33.58%) in 2016 [13]. The estimated number of smartphone users in Malaysia was 15.6 million (75.9%) in 2017, 78% in 2018, and this number is expected to swell by 20.9% in 2023 [14,15]. Users have a strong attachment and dependency to their smartphones with at least one out of four users constantly checking their phones even without notification (27.1%) [15].

Smartphones are predominantly seen as a promising and cost-effective medium to deliver health-related interventions [16]. To date, there are a few app-based interventions developed for unintentional home injuries; for example, Make Safe Happen for parents of children aged 0-12 years [11,17], Caregivers for children aged 0-6 years [18], Primary Caregivers of preschoolers aged 3-6 years [19,20], and Mothers with children under 3 years of age [21]. Education and promotion can be implemented through this platform to reach a larger segment of the population. With the rapid increase of smartphone usage in Malaysia and ease of access, a smartphone app intervention could be an effective solution for child injury prevention.

The novel idea of the ChildSafe app development came around as an experimental intervention designed to educate Malaysian parents and caregivers on child injury prevention at home. The ChildSafe app consists of 4 main features and functions: the home injury hazard assessment tool, interactive home safety tutorial, reminder feature addressing hazards identified during the assessment, and “tip of the week” feature. From the home injury hazard assessment tool, parents/guardians can identify and quantify existing hazards regarding childhood injury within the home as well as monitor changes occurring as a result of the interventions. The interactive home safety tutorial provides specific information on ways by which home injury hazard risks could be altered to make the environment safer for the child. The reminder feature serves as a feedback mechanism for parents/guardians to address the hazard and correct it. Tip of the week enhances the understanding of burden of childhood injury and knowledge on addressing it. At its nascent stage, acceptability of smartphone-based technology is important to be evaluated. Thus, a study was carried out to assess parents’ perception and acceptability of the app in terms of functionality, acceptability, and ease of use.

The consolidated framework for implementation research (CFIR), a conceptual framework that was established to guide systematic assessment of multilevel implementation to identify aspects that may affect implementation and effectiveness of an intervention, was used in this study. CFIR specifies 39 constructs acting as factors influencing intervention implementation and these are mapped into 5 major domains outlining potential barriers and facilitators of implementation or intervention outcomes: (1) *characteristics of an intervention*, (2) *outer setting* of the organization in which intervention is being implemented, (3) *inner setting* of the organization, (4) *characteristics of individuals* involved in the intervention, and (5) the *implementation process* [22]. With this framework in mind, this study aimed to evaluate acceptance of the ChildSafe smartphone app intervention by parents/guardians.

## Methods

### Study Design

This is a qualitative study assessing parental/guardian experience in using the ChildSafe smartphone app.

### Setting

The study was performed in the Sungai Buloh area, Selangor, Malaysia. This suburban area was selected as it fulfilled the inclusion criteria of a growing community that is neither too rural nor overly urban based on ease of access and high penetration of smartphone use. Inclusion criteria for participants were as follows: households must have at least one child between 0 and 59 months of age, parent/guardian is available as participant, and owned an Android-based device.

### Intervention

**Figure 1:**
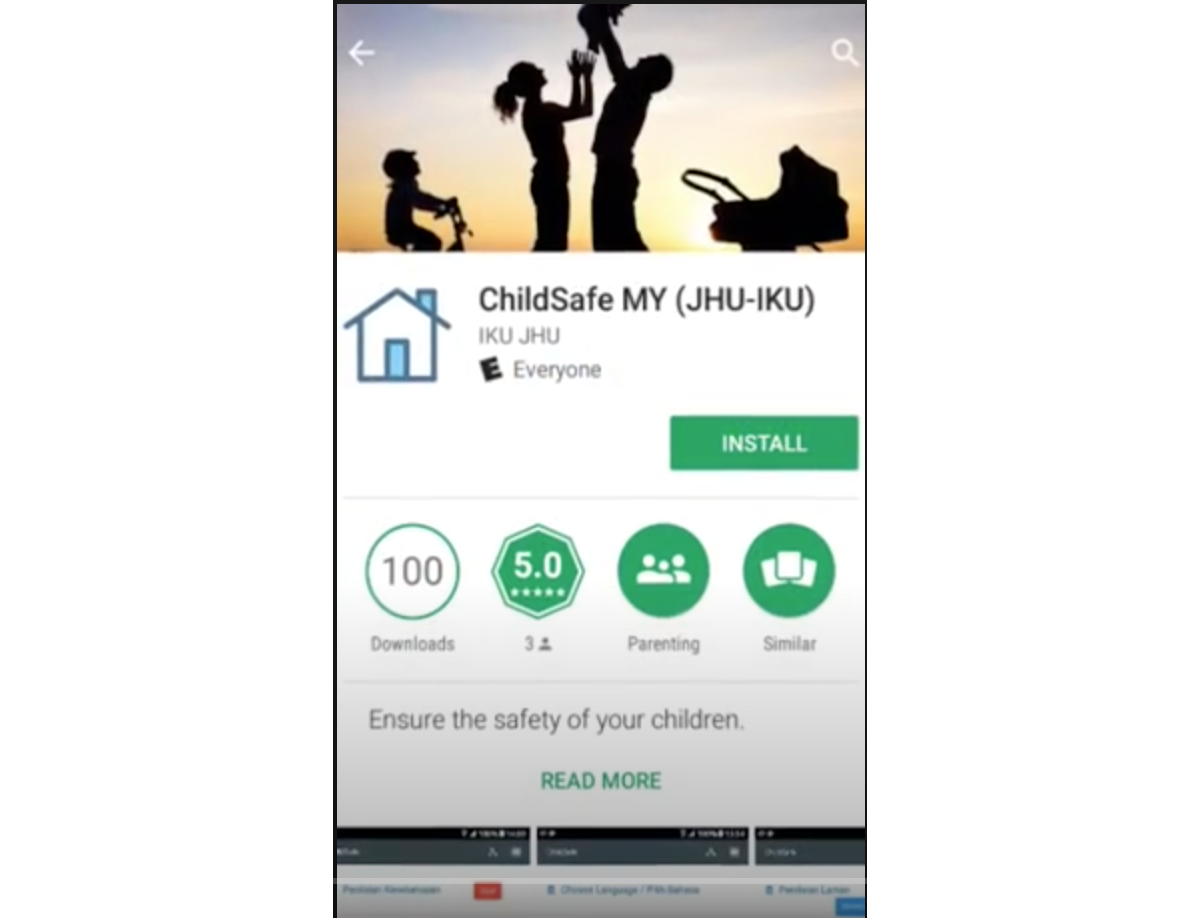
App download screen.

**Figure 2:**
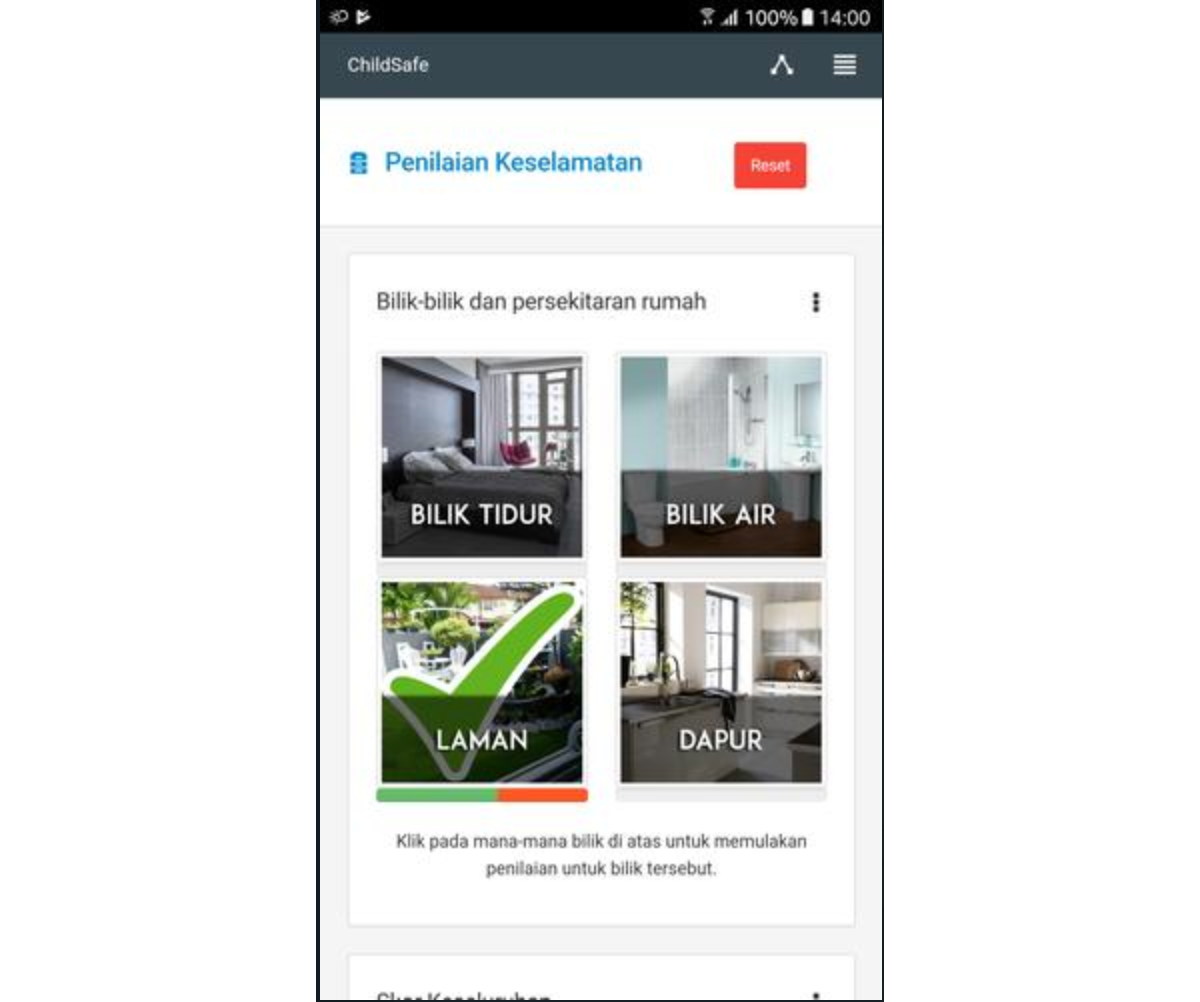
Safety assessment.

The ChildSafe app version 1.0 is a smartphone-based app designed to collect data and evaluate hazards risk reduction and prevention of child injuries at home, by focusing on feasible and measurable interventions within homes (Figure 1). The app was designed to map in-home risk of injuries among younger children (aged < 59 months), and intended to stimulate hazard reduction (Figure 2). The app is installed on the participants’ smartphone during the first visit. It has 4 main functions: a home injury hazard assessment tool, a home safety tutorial, a reminder feature for addressing hazards during the assessment, and tips on child safety. It is not publicly available, but for its use within this study a specific link to download the app was created.

### Study Participants

Study population included households with at least one child between the age of 0 and 59 months (Table 1). Participants were parents/guardians, able to read and converse in Malay or English, residing in the area of study during the study period, and fulfilled user app criteria of being either a complete user (completed tutorial and assessment), a partial user (completed at least tutorial but not assessment), or a nonuser (did not complete the tutorial or assessment). Participants were drawn from the main study (prospective cohort study) population and were identified by the main study research officers. A total of 27 parents/guardians participated. The number of participants was based on data saturation whereby the interview stopped at the 27th participant when there was no more new information based on the questions asked. All participants approached agreed to be interviewed and gave consent.

**Table 1:** Participants characteristics (N=158).

Characteristic				Value, n (%)
**Respondents’ relationship to the child**				
	Mother			118 (74.7)
	Father			36 (22.8)
	Others			4 (2.5)
**Gender of respondents**				
	Male			36 (22.8)
	Female			122 (77.2)
**Age group (years) respondents**				
	Under 30			29 (18.4)
	30-39			96 (60.8)
	40 and over			33 (20.9)
**Educational level**				
	Primary education and lower			4 (2.5)
	Secondary education			45 (28.5)
	Tertiary education			109 (69.0)
**House description**				
	**Type of accommodation**			
		Flat	22 (13.9)	
		Terrace single	18 (11.4)	
		Terrace double	118 (74.7)	
	**House ownership**			
		Own	120 (75.9)	
		Rented	38 (24.1)	

### Data Collection

Interviews were conducted approximately 2 months into the main study implementation (between November 2017 and March 2018). This was done on the expectation that home safety assessments were completed and possible changes were made to improve child safety at home. Interview questions were developed from the CFIR framework which was used to the assess implementation process of a new intervention. The flow of questions was developed with specific prompting questions to assess user experience on the ChildSafe app. The questions were designed in both English and Malay to ease communication with participants.

Interviews were conducted face-to-face by research team members who were trained in qualitative methods and not acquainted to any participants to avoid potential response bias. Each in-depth interview was conducted at participants’ home, lasted between 30 and 120 minutes, and was audio-recorded and transcribed verbatim. Confidentiality was ensured by removing participant identifiers from transcripts. Each transcript was cross-checked by the research team through audio listening and field notes.

### Data Analysis

Data were analyzed using a thematic analysis, manually coded, and arranged using MS Excel by research team members who had previous experience in conducting qualitative analysis, were familiar with CFIR, and experts in their own field. The first level of analysis employed an inductive approach whereby researchers read transcribed interviews to identify preliminary themes independently. Relevant text segments were selected concerning acceptability and experience in using the app. At this stage, the researcher identified similar patterns in the data and developed first-level coding themes that could explain those patterns. Consensus on first-level themes was gained from other research members as well. The text segments were sorted using the identified themes. At the second level the research members independently identified and matched the themes together with best representative quotes for each theme and mapped them with existing domains and constructs under CFIR. Through consensus with other research members, the themes were placed under the construct that was deemed most suitable, and with identification of these constructs, the final level of identifying the respective domain that encompasses the constructs was completed (Table 2). The quotes that best represent the domains and constructs chosen to support the results are presented in Multimedia Appendix 1.

**Table 2:** Perception of the ChildSafe Mobile App mapped on consolidated framework for implementation research domains.

Domain, constructs, and subconstructs				Perception on change		
				Positive presence		Negative presence
**Intervention characteristics**						
	Evidence strength and quality		✓			
	Relative advantage		✓			
	Adaptability		✓			
	Trialability		✓			
	Design quality and packaging		✓			
**Inner setting**						
	**Implementation climate**					
		Compatibility	✓			
		Relative priority	✓			
		Goals and feedback	✓			
	**Readiness for implementation**					
		Available resources	✓		✓	
		Access to knowledge and information	✓			
**Characteristics of individuals**						
	Knowledge and beliefs about the intervention		✓		✓	
	Self-efficacy		✓		✓	
	Individual stage of change		✓		✓	
	Other personal attributes		✓			

### Availability of Data and Materials

The data are not part of an online database but can be requested by writing to the Director of the Institute for Health Behavioral Research, National Institutes of Health, Ministry of Health Malaysia.

### Ethics Statement

This study was granted ethical approval by the Medical Research Ethics Committee of the Ministry of Health Malaysia [KKM/NIHSEC/P19-1450(12)].

## Results

### Overview

A total of 27 transcripts were analyzed. From the 5 domains under CFIR, 3 were applicable: intervention characteristics, inner setting, and characteristics of individuals.

### Intervention Characteristics

Of the 8 constructs in the intervention characteristics, 6 constructs which portrayed the app’s acceptability were identified.

#### Evidence Strength and Quality

Users believed the ChildSafe app, which was created to help parents and caregivers with young children, will make their home safer as they become more aware and were able to identify the potential hazards in and around their house. Users feel the importance of smartphones as one of the channels of information that helps disseminate safety tips on child injury prevention. Hence, they have also recommended the app to their families and friends. Additionally, users strongly recommended that the app should be promoted in governmental institutions, schools, health clinics, and nursery centers.

#### Relative Advantage (Observability)

With regard to the ease of availability and use of smartphones, most users approached owned one to stay connected, and thus the app was easily accessed wherever/whenever they need to check and refer about safety tips at their own pace. Users also shared that it is time saving compared with surfing information in social media and existing conventional media, and that it makes the dissemination of health information on child injury prevention easier and have wider coverage.

Apart from that, learning is made easier with the app, it engages the users to make their home safe, and it fulfills their needs on hands-on experiential learning related to the child injury issue. The app is considered a cost-effective medium to deliver the home safety information and covers a wide range of multiple topics in a single platform. Users also highlighted that the app served as their reference guide and reminder (reminder list on behavioral hazard for safety tips of the week).

#### Adaptability

The developers of the mobile health (mHealth) app have customized it to address the local language needs of users for better comprehension. Users acknowledged that the bilingual option (ie, users can select either English or Malay based on their preference) made them easy to understand and follow instructions on the app.

#### Trialability

Because of the easy accessibility of the mobile app, most users approached were willing to download the app on child injury prevention to try it out in order to know more about its purpose and usefulness, which subsequently will contribute to making a decision on whether the app is favorable or unfavorable to adopt.

#### Complexity

Users perceived the app easy to operate conceptually and technically, as users who owned a smartphone would have accustomed to the functionality of the device and the app.

#### Design Quality and Packaging

Most users expressed their satisfaction and shared their appreciation on the feasibility and usability of the app aesthetics when navigating it (clear layout; color, size, and font used; image using real pictures; easy or clear instruction; and a wide coverage of all areas of the house concerning potential hazards at home for them to cross check at all times).

On top of that, the app does not take up too much memory space of the phone. Participants also noted that there is an interactive home safety tutorial section embedded and a constant safety reminder feature (to-do list on environmental hazard) will appear once they switch on the app whenever they have not addressed the particular hazard as recommended.

### Inner Setting

#### Implementation Climate

##### Compatibility

Users perceived that because they have a smartphone and the installation process for the ChildSafe app is similar to that of other apps, it is fairly easy for them to understand and execute the task by taking safety precautions to prevent injuries to their children at home (social norms and values). Users expressed their enthusiasm to continue using the app as they found it rather convenient to learn about prevention of unintentional injuries. They strongly believed that the equipped knowledge and skills will help prevent unintentional injuries in their child as well as protect the child under their care.

##### Relative Priority

Users believed the app should be extended to have a wider coverage so that the community will become more aware of child safety at home and will benefit more. They also felt that this app is especially useful for parents, teachers, and caretakers.

##### Goals and Feedback

Users stated that the ChildSafe app will help them delve into child safety at home regardless of the level of knowledge they possess and increase their awareness on child safety at home. Users noted that the tips given via the app are very useful and that they can apply them to make improvements to their home to prevent unintentional injury. Based on the feedback from users, the objectives of the development of the mobile app have been achieved.

#### Readiness for Implementation

##### Available Resources

Because of the necessity and availability of smartphone in our daily lives, with most having at least one, the penetration of the ChildSafe app was higher, which made it easier to disseminate information on child injury at home. Users were eager to participate in the study as they want to ensure the safety of their children at home.

##### Access to Knowledge and Information

Most participants commented that the ChildSafe app was easy to use, understand, and do what is instructed to do. The instructions given allow users to think about what is important and useful, which may not have been thought of previously, especially for certain target populations. Users also felt that the educational information in the app is so comprehensive and more than what is offered in textbooks. The app indirectly engaged the users to spend some time checking each place in their home thoroughly before they can proceed to the next room.

#### Characteristics of Individuals

##### Knowledge and Beliefs About the Intervention

App information developed based on study and research is believed to be more reliable, trustable, and credible. In addition, the credibility of source is recommended by recruited staff members from the Ministry of Health. The app also acts as a preventive tool, as it presents a reminder of things to be put in place in every location of the house, thereby enabling users to be more aware of it. Because parents value the importance of their child’s safety, this app helped them identify the common dangers at home, based on which improvements were made.

##### Self-Efficacy

The app indirectly increased the confidence and influenced the parents to be more cautious and take precautions necessary to avoid injury in children. Among the measures taken are taking time to check thoroughly every corners of the house they may have missed out to ensure they are safe and buying the right toys for their children to play with and avoiding buying small toys that their children might put into their mouth.

##### Individual Stage of Change

The ChildSafe app has prompted the users on the receptiveness to change by making home improvement changes to avoid unforeseen unintentional injury among their children at home. With reference to the verbatim analyzed, most of the users have made changes within a short period and successfully maintained the recommended behavior changes.

##### Other Personal Attributes

The app served as a learning tool for the users, as they were excited to venture more given the score they have achieved, as well as created awareness among the users. Whenever users completed a task, they will be awarded a score that indirectly created interest and motivated them to go to the next stage. Users own capability and completeness in accessing the smartphone enabled them to use the mobile-based ChildSafe app without much issue.

## Discussion

### Principal Findings

The CFIR conceptual framework has provided an analytic lens of evaluation to determine the key success of the ChildSafe app implementation. The intervention study results provide an overview of (1) the users’ perceived characteristics of the innovation which will encourage them to use the ChildSafe app (behavioral intention), (2) the evaluation of the usability and feasibility of mHealth apps, (3) the innovation decision process that can facilitate the implementation of the app, and (4) the adoption phases that will lead to the adoption or resistance of the innovation.

Nilsen [23] has classified implementation frameworks into 5 categories: process models, determinant frameworks, classic theories, implementation theories, and evaluation frameworks. CFIR represents the determinant framework in this study to identify the confound factors that will contribute to the effectiveness of the interventions and their implementation [24], that is, evidence-based practice [25]. In line with the evidence-based practice, Damschroder et al [25] pointed out that in any intervention evaluation researchers must evaluate not only summative endpoint health outcomes but also the formative evaluation outlined in CFIR to assess the implementation effectiveness, the sustainability of the intervention, and dissemination of findings [25]. Dissemination and implementation studies particularly focus on the implementation impact to facilitate the adoption and implementation of the innovation or evidence-based intervention [26].

This ChildSafe app trial study is designed to evaluate the effectiveness of the parenting intervention in terms of the usability, feasibility, and acceptability (satisfaction on the innovation, eg, content or credibility) of parents and caregivers and their intention to reduce unintentional injuries among children at home [27].

Using CFIR and Diffusion of Innovation theory (proposed by Rogers) [28,29] as a guidance to predict adoption of mHealth apps, we assessed the users’ perceived characteristics of the innovation which will lead to the use of the ChildSafe app (behavior intention). Based on the findings of the study concerning the attributes of the *innovation characteristics* [30], our users have shown significant adoption of the mHealth app innovation. Users valued the app as a preventive and incremental (nonpreventive) innovation [31] (*relative advantage*) by making decisions to adopt, as it provides great advantages and benefits to manage their house from becoming an accident-prone site. In general, apps promoted by developers on a trial basis and those that are freely available will attract users willing to try them out (*trialability*). Furthermore, the reminder function of our app encouraged the users to check their surroundings on a frequent basis (*trialability*), as there will be a pop-up safety reminder on their smartphone screen when they turn on the app if they have not completed the checklist (self-monitoring). Most of the users perceived the app to be easy to understand and use (*complexity*). The availability of the app in bilingual language was well received by the users as they were able to choose either Malay or English for comprehension (*adaptability*). The *compatibility* of the app is relatively high for adoption as it is convenient to install because most users were already using smartphones and thus comfortable in downloading and installing apps. The portability of the smartphone makes it handy for the users to continue enjoying the mobile learning on the move [32] at a home setting (convenience of the mHealth intervention) [33] and navigate at any time (time saving).

Concerning the evaluation of mHealth app (*design quality and packaging*) quality criteria using the Mobile App Rating Scale (MARS) as proposed by Stoyanov et al [34,35], users relayed their satisfaction with the ChildSafe app. In terms of *engagement*, the app engaged the users by sending notification or reminder once they switch it on (*interactivity*) if they have not fully completed monitoring the things in each part of the house. Parents/caregivers (*target group*) also found the visual information for each part of the house together with the checklist and language used easy to understand. Users described that instructions via the app made them easy to understand and navigate (*functionality*) while the layout, suitability, and clear visuals (*aesthetics*) improved usability. Besides, the information presented is comprehensive and from a trustworthy source recommended by the Ministry of Health (*information quality*). Furthermore, the existing users’ recommendation to others on the benefit of this app and the frequency of their usage of the app (subjective quality of the app) enhanced the perceived impact of the app on the user’s knowledge, attitude, and intentions to address the targeted health behavior (ie, taking preventive measures to prevent unintentional injuries at home). Similarly, the app usability evaluation as addressed by Nielsen [36,37] depends on the 5 attributes: efficiency, satisfaction, learnability, memorability, and errors.

A full adoption and sustainment of the new innovation (ChildSafe app) is highly dependent on the 5 stages of the innovation decision process (ie, knowledge, persuasion, decision, implementation, and confirmation) [28], as suggested by Rogers. The first stage starts with *knowledge* (ie, access to information and knowledge and engaging), whereby the user is exposed to innovation, followed by *persuasion*, whereby the individual becomes interested and seeks further information about the innovation (affective). Self-efficacy (ie, one’s self-perception about his/her capability in using the app and his/her confidence in making his/her home a safe environment), as envisioned in Bandura’s social cognitive theory, plays a central role in successful behavior change. An individual’s belief is influenced by previous experiences, social persuasion, perception, and affective response to the behavior [38]. Supporting this view in the theory of reasoned action which highlights intention, attitude, and subjective norms will determine the individual’s intention to perform a behavior [39].

Interpersonal communication is used to spread word of the mouth concerning the testimonies on innovation usage (eg, network and communication/goals and feedback) and social reinforcement from peers (social system). This is within the adoption process that also contributes to the facilitation of the implementation effectiveness. The *decision* and *implementation* stages concerning the adoption of the innovation (readiness for implementation and executing) are determined by 2 predictors of attitude toward the usage, as indicated in the Technology Acceptance Model [40] on the perceived usefulness and perceived ease of use. Time of adoption also plays a crucial element in the adoption or resistance of the innovation [41]. Kaminski [42] pointed out that in Rogers’ Diffusion of Innovation theory, the adoption phases are highly dependent on the 5 types of adopters, namely, *innovators* (technology enthusiast), *early adopters* (visionaries), *early majority* (pragmatist), *late majority* (conservative), and lastly the *laggards* (sceptic). Rogers’ proposed phases are also comparable with the 5 stages of change in the Prochaska’s Transtheoretical Model of Behavior Change [43], namely, the *precontemplation* (not thinking of changing), *contemplation* (aware and thinking about changing), *preparation* (take necessary steps to change), *action* (making changes within a short period), and *maintenance* (successfully maintain the behavior changes) [39]. The confirmation stage reflects on either the facilitation or hindrance of ChildSafe app usage (reflecting and evaluating).

These stages are also consistent with the scale criteria checklist (App Behavior Change Scale [ABACUS]) suggested by McKay [44] to assess the potential of an app in encouraging behavior change. ABACUS involves assessment of knowledge and information, goals and planning, feedback, and monitoring and actions [45]. Proctor et al [46] proposed 8 outcomes for evaluation: *acceptability* (user satisfaction with various aspects of the innovation, eg, content, complexity, and credibility), *adoption* (uptake, utilization, intention to try), *appropriateness* (compatibility, usefulness), *feasibility* (suitability for use), *fidelity* (adherence), *implementation cost* (cost–benefit), *penetration* (coverage access), and *sustainability* (maintenance) [45-47].

In particular, due to the rapid growth of technology, technology-assisted communication devices have been vastly utilized to deliver and enhance health interventions. Hence, there was some promising evidence from Omaki et al [48] that technology-assisted communication devices are indeed effective in improving knowledge, awareness, and creating favorable responsible actions pertaining to prevention of unintentional injuries. With regard to the study by Jabaley et al [49], using an iPhone (mobile device) as an in-home child safety intervention among 3 families with young children reduced home hazards to almost zero [46]. Similar studies on injury prevention apps for parents and caregivers have also been undertaken by Roberts et al [17], McKenzie et al [11], Chow et al [21], and Ning et al [18-20], and these indicated that such apps have improved parental safety knowledge, environmental modification, and successfully facilitated behavior change.

### Strengths and Limitations

This study provides an underlying view on the importance of incorporating an evidence-based theoretical framework model and a health behavior change theory as a foundation when developing mHealth apps and designing mHealth interventions. CFIR itself is able to identify factors that determine the likelihood of implementing recommendations given by the app, its impact, and its effectiveness in behavior change to prevent or reduce unintentional home injuries in children, which in turn allows for future improvements to be made to the app to ensure all aspects of the framework are addressed for the benefit of the users.

The main limitation of the study pertain to the differences in cultural aspects. As the app was originally developed for Western use, some applicability does not resonate with the Malaysian population, which caused the users to feel disconnected when giving feedback. These included fire alarms, differences in toilet bowl designs, the absence of bathtubs in the Malaysian home, which in the Western countries are important elements in the assessment. Future studies may need to include a situational analysis or feasibility study before the start of the actual study.

**Recommendations**

The findings warrant future research studies to explore, examine, and provide insights into the potential areas for improvement on the developmental aspects with regard to the ChildSafe app:

- The study should be extended to the community and made available in different languages such as Mandarin and Tamil to reach out to different communities.
- Further improvement on the app coverage is necessary by incorporating missing features such as first aid; information on relevant emergency contact; setting reminders to check for functionality of safety devices; feedback; discussion forum to exchange opinions or ideas related to safety at home; teleconsultation or advice by experts or pediatricians for parents, caregivers, and teachers in elementary, primary, and secondary school; and interactive learning such as videos, interactive games, animation, and quiz not only for parents but also for children to learn; as well as providing regular updates to the information on the app, so that it can serve as an information hub and learning platform for all. In addition, improvisation of the tailored home safety information necessary to prevent injury in children and information on various safety aspects outside the house should be added to the app.
- Whether the app usability and feasibility in the particular community have improved the parents’ knowledge, awareness, and self-empowerment to take responsibility for their child’s health and safety should be investigated by examining the incidence rate of injury (epidemiological data) or by performing a comparison study between the intervention group and the control group during the duration of the study.
- A replicate study on the pretest of the app intervention in different community settings and population to assess the usability, acceptability, and feasibility is necessary.

### Conclusions

Despite being a prototype and lacking advanced features, the ChildSafe app has been perceived as a very useful tool by the participants and has induced behavioral changes in users, such as taking actions to improve their living quarters to reduce unintentional injuries for their children at home. Although some issues highlighted warrant the need to improve future versions of the app, the study has indicated the possibilities and usefulness of app-based interventions for the Malaysian setting. The results of this study can be used as a guide to assist the relevant multidisciplinary policy makers to address the three “Es” of interventions (education, enforcement, and engineering), as proposed in the Haddon matrix, to prevent unintentional injuries [2] on probably a larger scale should the improved app be made available publicly.

## Appendix

Multimedia Appendix 1 Example quotes organized thematically and mapped to CFIR domain and construct.

## Abbreviations

ABACUS: App Behavior Change Scale
CFIR: consolidated framework for implementation research
MARS: Mobile App Rating Scale
MDGs: Millennium Development Goals
SDGs: Sustainable Development Goals
U5MR: under 5 mortality rate
UNCRC: United Nations Convention on the Rights of a Child
