# Supplementary material for: Perceptions and Acceptability of a Smartphone App Intervention (ChildSafe) in Malaysia: Qualitative Exploratory Study
Source: JMIR Pediatr Parent. 2021 Jun 1;4(2):e24156. doi: 10.2196/24156 (PMC8207251; doi:10.2196/24156)
Supplement: Multimedia Appendix 1 [file pediatrics_v4i2e24156_app1.docx]

| Domain | Construct | Sub-Construct | Quotes |
| --- | --- | --- | --- |
| Intervention Characteristics | Evidence Strength and Quality |  | “this (app) is helpful…give awareness for parents to do right things in the house for the child safety… it serves as a reminder….a huge reminder and good…we just do it and follow.” [M1, Father, 32 years old] |
|  |  |  | “…….. think this app is good but for a parent that do not take care of their children every single time because they are working, they will think that this app is not really practical to them. When I went to my sister in law’s house last time, I reminded her to take care every single corner at her home (laughing) as they never care about the child’s safety at home before this. I have suggested my friends to use it also. I told them, if you want to know whether your home is safe for children or not, try use this apps. The house should be made safe for children.” [F10, Mother, 32 years old] |
|  |  |  | “how and where…..okay , in order to market this app widely, we can promote it among government institution, examples such as school, clinic, nursery. So from there, this can spread for general knowledge.” [F1, Mother, 35 years old] |
|  | Relative Advantage (Observability) |  | “easy because everything is in there. Tips always there, example safety in toilet, when we press the kitchen, tips will appear….haah easy to open, no need to search. Since the app inside the hand phone, if we want to read, we just use it. let say today we want to read about kid one year old, we open in one year time. maybe we already know, we see for infant from 0 to 7 months, how to take care of them.” [F6, Mother, 32 years old] |
|  |  |  | “eh no, Facebook and Instagram take a lot of my time.” [F8, Mother, 34 years old] |
|  | Adaptability |  | “the language is easy to understand.... the language is bilingual, we can (choose either) English or Malay language. Languages used are simple language”. [M2, Father, 37 years old] |
|  | Trialability |  | “so, when they come to my house, they explain this is for child health and safety. I really want to know about it.” [M3, Father, 40 years old] |
|  | Complexity |  | “currently, the app is easy to use even for beginner in IT.” [F12, Mother, 42 years old] |
|  | Design Quality & Packaging |  | “Okay, for me, so far, for which I remember using it, it has that ... Background with which it wants to highlight, can differentiate the font. The point is clear. That means if the background black, the writing that maybe in the color. Different colors also.” [M2, Father, 37 years old] |
|  | Design Quality & Packaging |  | “because in the house, it covers all, so it is easy to understand, it also has safety features and things to look into in the hall, kitchen, room, it is easy to check from there.” [F5, Mother, 43 years old] |
|  | Design Quality & Packaging |  | “(app) do not disturb (phone) in term of the app utilization…I am IT person, in term of app usage, it does not take up the memory or the phone processor.” [M1, Father, 32 years old] |
| Inner Setting | Implementation Climate | Compatibility | “…because this app easy to download in app store or google store. Then, everyone use hand phone, so it will always update. for example, if there is notification, people will quickly update; if this month, not yet updated. we can see what other safety features, (potential) injury to the children, so it is very good. it is impossible to say nowadays no one has hand phone, everyone will use hand phone.” [F3, Mother, 28 years old] |
|  |  | Relative Priority | “…because nowadays parents do not stay long with their children, do not sit for long, most of the children’s time spent in school, nursery...thus, those group must have some knowledge about this issue ….. in school, school teacher, the caretaker in nursery.” [M4, Father, 33 years old] |
|  |  | Goals & Feedback | “alhamdulillah, so far when I use the app, there is no problem, because it gives many examples such as in the toilet, how to prevent kid from drowning, or upside down buckets, I think all the information is useful. For myself, I just know that I have to place the bucket upside down after using it. for us, adult, if we don't have kids, we will fill the bucket with water because it is easy to use in the toilet. however, if we have kids, we have to empty or left very little amount of water inside it. the information is very good, example what should have in the room, what type of carpet, things that we should look out to prevent child injury. so, I think the information is good.” [F3, Mother, 28 years old] |
|  | Readiness for Implementation | Available Resources | “because this app easy to download in app store or google store. Then, everyone use hand phone, so it will always update. for example, if there is notification, people will quickly update; oh this month, not yet updated. we can see what other safety features, (potential) injury to the children, so it is very good. it is impossible to say nowadays no one had hand phone, everyone will use hand phone.” [F3, Mother, 28 years old] |
|  |  | Access to knowledge & information | “When I look at the app I can see what it wants, so I get to think about the safety in the house, what I need to arrange and keep those things that I cannot put everywhere… so for me, it is more to give me awareness for myself about the things inside my house… that could lead to child injury…” [M1, Father, 32 years old] |
|  |  |  | “this information is much more than textbook…this one also okay (laugh)…so like that laa…if go other houses, maybe can also get knowledge.” [M4, Father, 33 years old] |
| Characteristics of Individuals | Knowledge & beliefs about the intervention |  | “if the sources from Ministry of Health, of course it is trusted and reliable, but if from other sources, it is difficult to trust.” [F12, Mother, 42 years old] |
|  |  |  | “this app shows specific area such as kitchen, where and what are the dangerous things in kitchen such as how to keep the cooking oil, so we have to follow it. The same goes to living room and toilet. When I have this app, I can know which things is dangerous, where to put it, so I become more aware.” [F7, Mother, 40 years old] |
|  | Self-efficacy |  | “Almost half an hour because when I do it, I have to go check my bedroom. I do not simplify just clicking it. So still I look into it by identify whether it is ok or not or maybe some of it that I already know then I just do next question.” [F11, Mother, 37 years old] |
|  |  |  | “But when we have a child and start to buy a toy, we do not buy a small toy like Lego.” [F18, Mother, 31 years old] |
|  | Individual stage of change |  | “when I look at the app can read the info, so I have to think…(silent for a while)…about safety in the house, what I need to arrange and keep those things that cannot be put everywhere…so for me, it is more to give awareness for myself about things inside the house.....that can lead to child injury.” [M1, Father, 32 years old] |
|  | Other Personal Attributes |  | “if possible, I want to continue until the end…if it is fast, there is no problem…. eat what's there (refer to the food served by his wife) …. if there is no problem, we want to see until finish, because it looks interesting because we can know our score so, we can improve ourselves…the app is good, it shows Room 1, Room 2, it is good.” [M4, Father, 33 years old] |
|  |  |  | “happy. when I look at the chart, I am the highest.” [F7, Mother, 40 years old] |
